# *BRCA1/2* Polymorphisms and the Risk of Recurrent Implantation Failure in Korean Women

**DOI:** 10.3390/ijms272110032

**Published:** 2026-11-09

**Authors:** Hyeon Woo Park, Da Hwan Kim, Ji Hyang Kim, So Yeon Shin, Ji Eun Ko, Nam Keun Kim

**Affiliations:** 1 Division of Life Sciences, College of Life Sciences, CHA University, Seongnam 13488, Republic of Korea; wjphw1@gmail.com (H.W.P.); soda00831kr@naver.com (D.H.K.); 2 Department of Life Science, Graduate School, CHA University, Seongnam 13488, Republic of Korea; 3 Department of Neurology, CHA Bundang Medical Center, School of Medicine, CHA University, Seongnam 13496, Republic of Korea; 4 Department of Obstetrics and Gynecology, CHA Bundang Medical Center, School of Medicine, CHA University, Seongnam 13496, Republic of Korea; bin0902@cha.ac.kr (J.H.K.); syshin88@chamc.co.kr (S.Y.S.); a186009@chamc.co.kr (J.E.K.)

**Keywords:** recurrent implantation failure, *BRCA1*, *BRCA2*, single-nucleotide polymorphism, rs1799943, DNA double-strand break repair, haplotype, gene–gene interaction

## Abstract

Recurrent implantation failure (RIF) is a major cause of unsuccessful assisted reproduction, yet its genetic basis remains poorly defined. Because *BRCA1* and *BRCA2* are central to homologous recombination-mediated repair of DNA double-strand breaks, we examined whether common variants in these genes are associated with RIF. Seven *BRCA1/2* single-nucleotide polymorphisms, prioritized from whole-exome sequencing data, were genotyped in 128 women with RIF and 330 controls with proven natural fertility, and associations were estimated by age-adjusted logistic regression with false discovery rate (FDR) correction. The *BRCA2* rs1799943 A allele was associated with lower odds of RIF under the heterozygous (adjusted odds ratio [AOR] 0.445, FDR *q* = 0.006) and dominant (AOR 0.443, *q* = 0.004) models; no other variant remained associated after correction. In exploratory analyses of the same participants, this inverse association was largely confined to carriers of the *BRCA1* rs12516 A allele (interaction *q* = 0.003), the *BRCA2* haplotype carrying the rs1799943 A allele was associated with lower odds of RIF (AOR 0.551, *q* = 0.012), and nominal associations of genotype combinations involving rs15869 were not supported by formal interaction testing. These findings identify rs1799943 as a candidate variant associated with RIF that warrants replication and functional investigation.

## 1. Introduction

Infertility has emerged as a major public health concern worldwide, and its impact is particularly severe in South Korea [1], where the total fertility rate fell to a record low of 0.72 in 2023 [2]. Recurrent implantation failure (RIF) refers to the failure to achieve a clinical pregnancy after the repeated transfer of morphologically good-quality embryos during in vitro fertilization (IVF) [3,4,5]. Despite advances in assisted reproductive technology, approximately 10% of women undergoing IVF experience RIF. Factors implicated in RIF include embryo aneuploidy, inadequate endometrial receptivity, immune imbalance, endocrine dysregulation, thrombophilia, and suboptimal vascular development [5,6,7]. Morphological grading does not guarantee chromosomal normality, and a substantial proportion of morphologically good embryos are aneuploid, particularly with increasing maternal age [8,9]; implantation outcomes therefore depend on both embryonic competence and maternal determinants such as endometrial receptivity and embryo–uterine signaling [10,11]. Polymorphisms in hormone-related genes such as *FSHR*, *INHA*, *ESR1*, and *BMP15* have been reported to be associated with RIF in Korean women [12]; however, the contribution of genes that maintain genomic integrity to RIF susceptibility remains largely unexplored.

**Figure 1:**
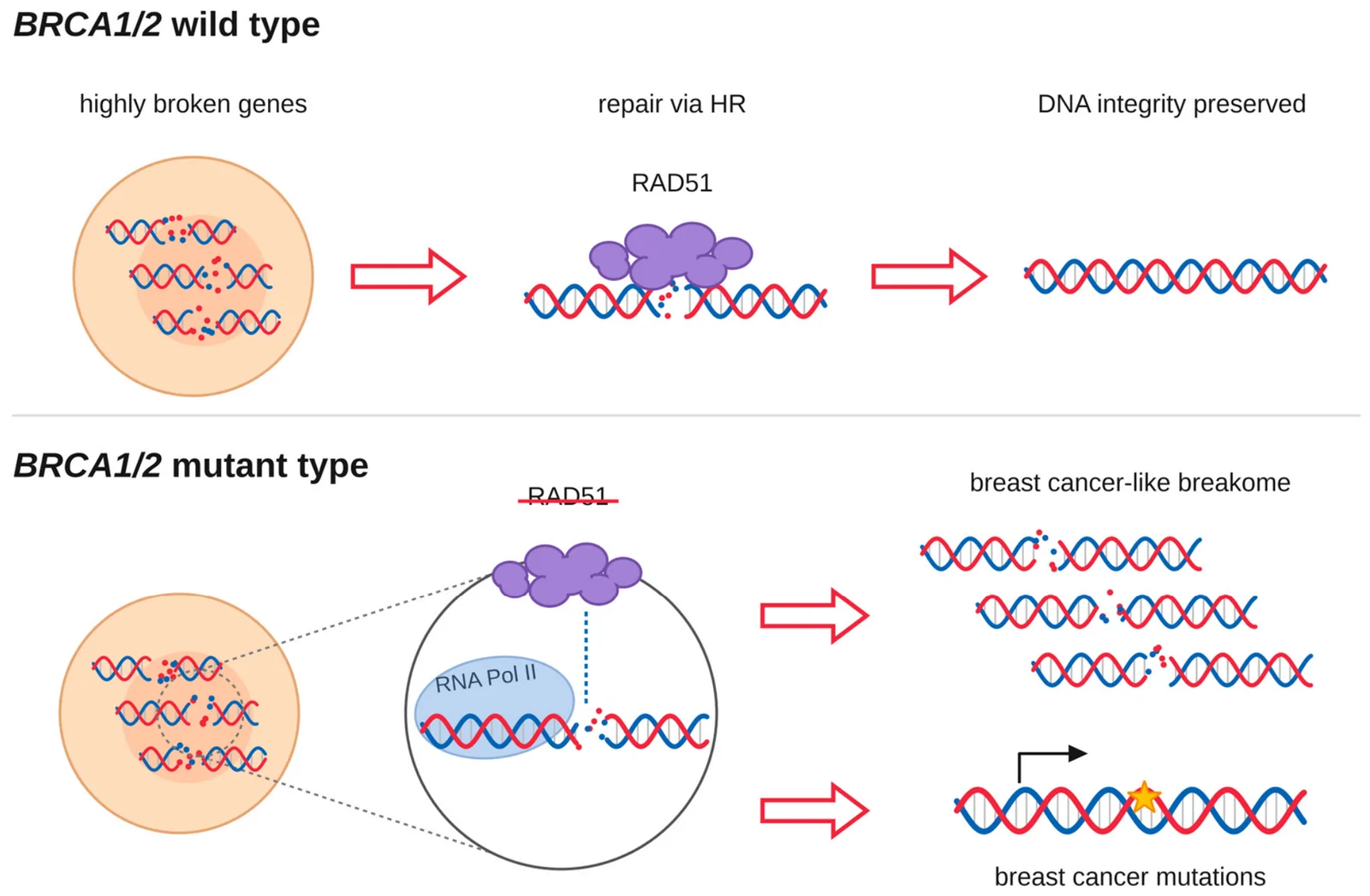
*BRCA1/2* and DNA double-strand break (DSB) repair. (**Top**) In cells with functional BRCA1/2, DSBs in highly broken genes are repaired by RAD51-dependent homologous recombination (HR), preserving DNA integrity. (**Bottom**) When BRCA1/2 function is impaired, RAD51 is not efficiently recruited to the break site, and DSBs arising at actively transcribed loci occupied by RNA polymerase II (RNA Pol II) persist, generating a breast cancer-like “breakome” and promoting the accumulation of cancer-associated mutations. Based on the model described in [22].

DNA double-strand breaks (DSBs) arise physiologically during oogenesis, meiotic recombination, and follicular maturation and may also accompany the rapid cell proliferation and decidualization of the peri-implantation endometrium, and they must be accurately repaired to preserve genomic stability. *BRCA1* and *BRCA2*, originally identified in the mid-1990s as major tumor-suppressor genes underlying hereditary breast and ovarian cancer [13,14], play indispensable roles in homologous recombination (HR)-mediated DSB repair [15,16,17]. In response to DSBs, BRCA1 is phosphorylated in an ATM-dependent manner, which is required for an appropriate cellular response to these lesions [18], and DNA damage induces its relocalization, together with BARD1 and RAD51, to sites of DNA replication [19]; in complex with BARD1, BRCA1 also functions as an E3 ubiquitin ligase [20]. BRCA2 acts downstream of damage recognition, promoting the loading of RAD51 recombinase onto single-stranded DNA and stabilizing RAD51 nucleoprotein filaments, thereby stimulating RAD51-mediated recombination [21]. Impairment of this pathway has direct genomic consequences: genome-wide mapping of DSBs in non-malignant mammary epithelial cells from carriers of *BRCA1/2* pathway mutations showed that breaks accumulate at highly expressed genes, many of which are frequently mutated in breast cancer, generating a cancer-like “breakome” before malignant transformation (Figure 1) [22].

Although *BRCA1/2* were first characterized in the context of oncogenesis, accumulating evidence indicates that their functions extend to female reproductive physiology. In human oocytes, the expression of BRCA1 and of other ATM-mediated DSB repair genes declines with age, and *Brca1*-deficient mice show reduced primordial follicle reserve, impaired reproductive capacity, and increased accumulation of DSBs in the remaining follicles [23]; oocytes with unrepaired DSBs are eliminated through a CHK2-dependent DNA damage checkpoint [24]. Clinically, women carrying pathogenic *BRCA1/2* variants exhibit a poorer response to ovarian stimulation, diminished ovarian reserve, lower anti-Müllerian hormone levels, and earlier menopause [25,26,27,28,29]. Biallelic *BRCA1* and *BRCA2* variants have recently been described in patients with isolated diminished ovarian reserve or primary ovarian insufficiency without cancer or the typical clinical features of Fanconi anaemia [30,31], and genes originally linked to cancer susceptibility are increasingly recognized as contributors to unexplained female infertility [32].

Beyond the oocyte, BRCA1 interacts with steroid hormone signaling, which governs endometrial proliferation and decidualization; for example, BRCA1 inhibits estrogen receptor-α-mediated transcription [33]. However, these observations derive largely from cancer cell models, and direct evidence for a role of BRCA1/2 in human endometrial receptivity is limited. Whether common, non-pathogenic *BRCA1/2* variants influence reproductive outcomes has not been established. Variants within microRNA target sites and other untranslated regions can modify post-transcriptional regulation [34], and the *BRCA1* 3′ untranslated region (UTR) variants rs8176318 and rs12516 have been reported to be functional or to correlate with *BRCA1* mRNA expression [35,36], providing a plausible, although unproven, mechanism by which such polymorphisms could modulate DNA repair capacity in reproductive tissues.

**Figure 2:**
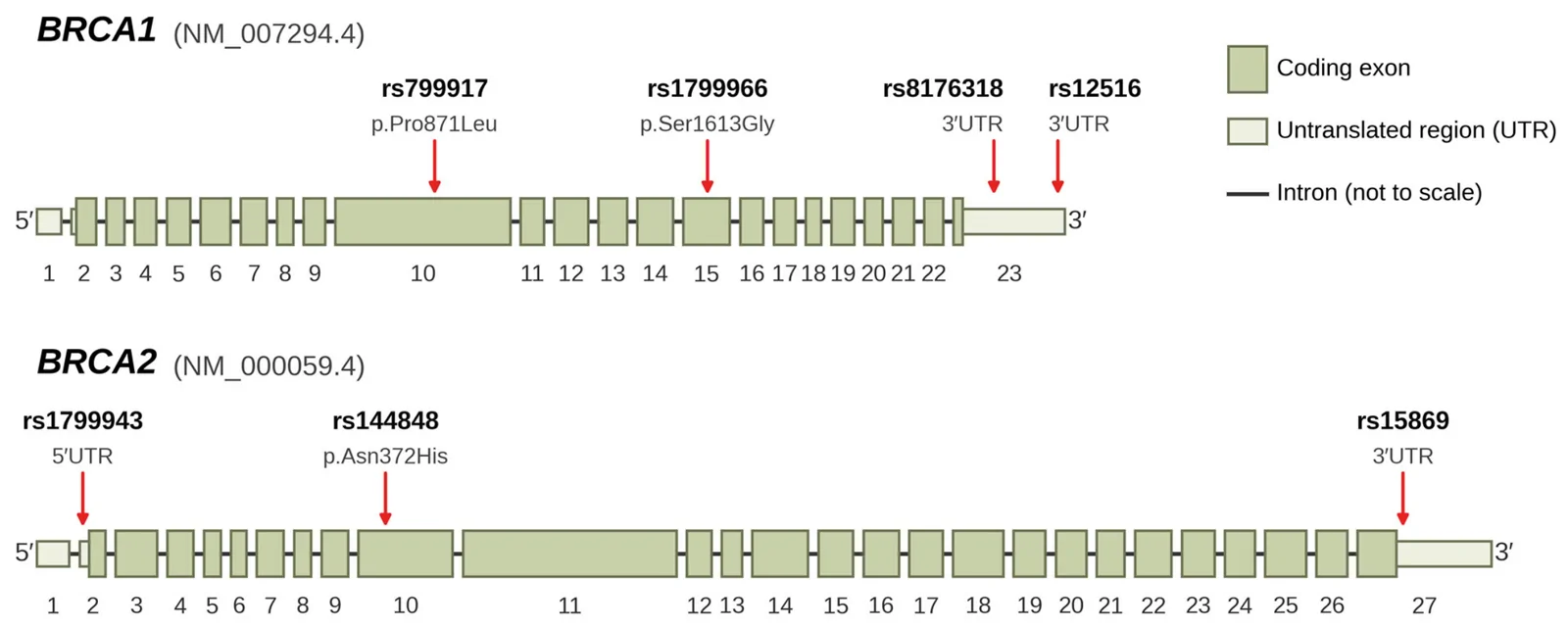
Genomic locations of the seven *BRCA1/2* polymorphisms analyzed in this study. Exons are shown as numbered boxes, with coding regions in dark boxes and untranslated regions (UTRs) in light, narrower boxes; introns are shown as lines and are not drawn to scale. Red arrows indicate the positions of the selected variants. In *BRCA1*, rs799917 (exon 10, p.Pro871Leu) and rs1799966 (exon 15, p.Ser1613Gly) are missense variants, whereas rs8176318 and rs12516 are located in the 3′ UTR of exon 23. In *BRCA2*, rs1799943 is located in the 5′ UTR of exon 2, rs144848 (exon 10, p.Asn372His) is a missense variant, and rs15869 is located in the 3′ UTR of exon 27. This figure is original and was drawn by the authors using exon and variant coordinates retrieved from the Ensembl database (GRCh38) for the MANE Select transcripts NM_007294.4 (*BRCA1*) and NM_000059.4 (*BRCA2*); exons are numbered according to current HGVS nomenclature.

Despite this biological rationale, no study to date has examined whether common *BRCA1/2* polymorphisms are associated with RIF. Using whole-exome sequencing (WES) data generated by our group from women with primary ovarian insufficiency (POI) and women with RIF, together with fertile controls, and a previously described variant-discovery pipeline [37], we prioritized seven *BRCA1/2* SNPs (*BRCA1* rs12516, rs1799966, rs799917, and rs8176318; *BRCA2* rs144848, rs15869, and rs1799943; Figure 2) that differed in allele frequency between patients and controls in both WES datasets and that are common (minor allele frequency ≥ 0.05) in Korean reference panels. In the present study, we genotyped these seven SNPs in a separate case–control cohort of Korean women with RIF and fertile controls. Single-locus associations were evaluated as the primary analysis, and genotype-combination, interaction, haplotype, and allele-combination analyses were performed as exploratory analyses. Our aim was to examine whether common variation in genes of the BRCA-mediated genome-maintenance pathway is associated with RIF.

## 2. Results

### 2.1. Clinical Characteristics of the Study Participants

The baseline characteristics of the 128 women with RIF and 330 fertile controls are summarized in Table 1. Mean age (34.06 ± 3.16 vs. 33.44 ± 4.05 years, *p* = 0.112) and body mass index (21.39 ± 3.26 vs. 21.66 ± 3.37 kg/m^2^, *p* = 0.533) did not differ between groups. Serum follicle-stimulating hormone concentrations were comparable (*p* = 0.940), whereas luteinizing hormone (*p* = 0.008), estradiol (*p* = 0.039), and thyroid-stimulating hormone (TSH; 2.16 ± 1.38 vs. 1.58 ± 1.12 µIU/mL, *p* < 0.001) were higher in the RIF group, although mean TSH values in both groups were within the reference range. Coagulation indices (prothrombin time [PT] and activated partial thromboplastin time [aPTT]), renal function markers, total cholesterol, and several hematological parameters also differed between groups, whereas homocysteine, folate, glucose, and uric acid did not. Because clinical measurements were only available for subsets of participants and the timing of blood sampling relative to the menstrual cycle, fertility treatment, and, for controls, pregnancy was not recorded, these between-group differences should be interpreted with caution.

### 2.2. Association of BRCA1/2 Polymorphisms with RIF

All seven *BRCA1/2* polymorphisms were successfully genotyped in every participant, and genotype distributions in the control group conformed to Hardy–Weinberg equilibrium (all *p* > 0.05; Table 2). Single-locus association testing of the seven SNPs under four genetic models (28 tests) constituted the primary analysis, and multiplicity was controlled using the Benjamini–Hochberg false discovery rate (FDR) [38]. Among the four *BRCA1* variants (rs12516 G>A, rs1799966 T>C, rs799917 G>A, and rs8176318 C>A), none was associated with RIF; the rs12516 GA + AA genotypes showed a non-significant trend toward higher odds of RIF in the dominant model (adjusted odds ratio [AOR] 1.435, 95% confidence interval [CI] 0.948–2.171, *p* = 0.087, *q* = 0.612). The *BRCA2* rs144848 A>C and rs15869 A>C variants likewise showed no significant single-locus association.

In contrast, the *BRCA2* rs1799943 G>A polymorphism was associated with RIF. The GG genotype was more frequent among RIF patients than among controls (50.8% vs. 30.9%). Relative to the GG genotype, the GA genotype (AOR 0.445, 95% CI 0.284–0.698, *p* < 0.001, *q* = 0.006) and carriage of the A allele under the dominant model (GA + AA vs. GG; AOR 0.443, 95% CI 0.291–0.674, *p* < 0.001, *q* = 0.004) were associated with lower odds of RIF, and both associations remained significant after FDR and Bonferroni correction. The AA genotype showed a nominal association (AOR 0.449, 95% CI 0.237–0.850, *p* = 0.014) that did not remain significant after FDR correction (*q* = 0.130), and the recessive model was not significant (AOR 0.671, 95% CI 0.370–1.218, *p* = 0.190). The allele-based analysis yielded a consistent result (A vs. G allele: odds ratio [OR] 0.580, 95% CI 0.427–0.788, *p* < 0.001; Table S1). In sensitivity analyses (Table S2), additional adjustment for body mass index (BMI) or TSH changed the estimates only slightly relative to the age-adjusted estimates in the same subsets (GA + AA vs. GG: AOR 0.258 vs. 0.262 in the subset with BMI data and 0.712 vs. 0.665 in the subset with TSH data), and the association persisted after exclusion of participants with TSH > 4.5 µIU/mL (AOR 0.473, 95% CI 0.309–0.724). However, the estimate in the subset with TSH data (130 controls and 102 patients) was attenuated and not statistically significant.

### 2.3. Exploratory Genotype-Combination and Interaction Analyses

As an exploratory analysis, all pairwise genotype combinations of the seven SNPs were compared between RIF patients and controls, with the combination homozygous for the major allele at both loci as the reference (142 comparisons; complete results in Table S3). Thirteen combinations showed nominal associations with RIF (*p* < 0.05; Table 3), but none remained significant after FDR correction (all *q* ≥ 0.25). The six nominally significant combinations that included the rs1799943 GA or AA genotype were each associated with lower odds of RIF (AOR 0.369–0.473), consistent with the single-locus association, although this was not the case for every rs1799943 A-containing combination (Table S3). Conversely, the *BRCA1* rs12516 GA/*BRCA2* rs1799943 GG (AOR 2.698, 95% CI 1.338–5.437) and rs12516 AA/rs1799943 GG (AOR 3.770, 95% CI 1.210–11.743) combinations were associated with higher odds of RIF. Several combinations involving the rs15869 AC or CC genotype also showed nominally elevated AORs, but these estimates were based on fewer than five individuals in at least one group (e.g., rs144848 AC/rs15869 CC: six patients and two controls) and had wide confidence intervals.

To determine whether these patterns reflected statistical interaction, multiplicative SNP × SNP interaction terms were tested in age-adjusted logistic regression models for all 21 SNP pairs under log-additive and dominant codings (Table S4). No interaction involving rs15869 remained significant after FDR correction (all *q* ≥ 0.074); thus, the nominal associations of rs15869-containing combinations were not supported by formal interaction testing. In contrast, significant interactions under the log-additive model were observed between *BRCA2* rs1799943 and the *BRCA1* variants rs12516 (ratio of ORs 0.401, 95% CI 0.241–0.667, *p* for interaction <0.001, *q* = 0.003), rs1799966 (0.405, 95% CI 0.241–0.678, *q* = 0.003), and rs8176318 (0.435, 95% CI 0.263–0.718, *q* = 0.004), three variants in strong LD with one another. In stratified analyses, the rs1799943 A allele (GA + AA vs. GG) was associated with lower odds of RIF among carriers of the *BRCA1* rs12516 A allele (AOR 0.227, 95% CI 0.121–0.425, *p* < 0.001) but not among rs12516 GG homozygotes (AOR 0.756, 95% CI 0.420–1.362, *p* = 0.352), with similar patterns for rs1799966 and rs8176318 (Table S5). Interaction terms for *BRCA1* SNP pairs within the same LD block mainly reflect haplotype structure, and several were not estimable because of sparse discordant genotype categories; these pairs were therefore evaluated by haplotype analysis (Section 2.4).

### 2.4. Linkage Disequilibrium, Haplotype, and Allele-Combination Analyses

**Figure 3:**
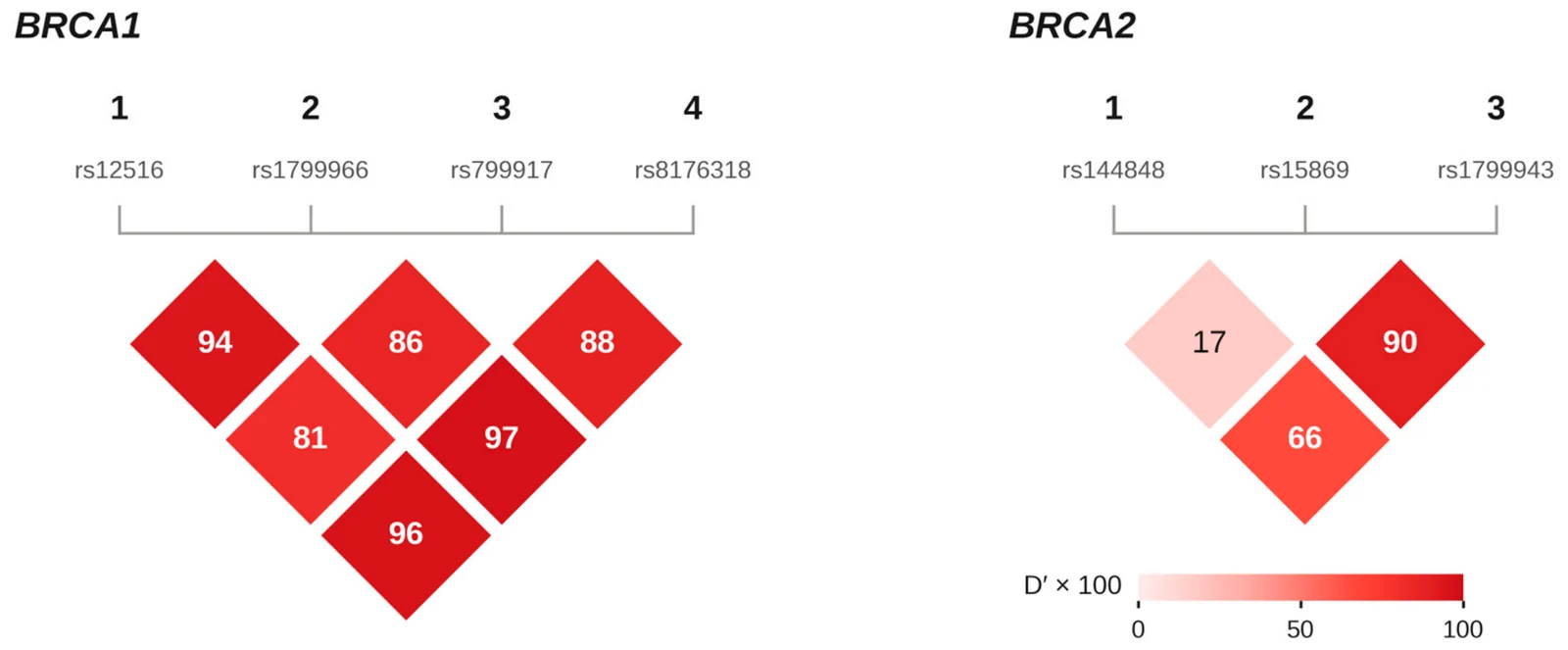
Linkage disequilibrium (LD) patterns of the seven *BRCA1/2* polymorphisms in all study participants (*n* = 458). LD was estimated separately within each gene; pairwise values are expressed as D′ × 100 and are represented by color intensity, with darker red indicating stronger LD. *BRCA1*: 1, rs12516; 2, rs1799966; 3, rs799917; 4, rs8176318. *BRCA2*: 1, rs144848; 2, rs15869; 3, rs1799943.

Pairwise linkage disequilibrium (LD) among the seven polymorphisms, estimated within each gene in all study participants, is shown in Figure 3. The four *BRCA1* variants were in strong LD with one another (D′ × 100 ≥ 81), consistent with their location within a single haplotype block. Within *BRCA2*, rs15869 and rs1799943 were tightly linked (D′ × 100 = 90), whereas rs144848 showed only weak-to-moderate linkage with the other two *BRCA2* variants (D′ × 100 = 17 and 66). Because *BRCA1* and *BRCA2* are located on different chromosomes (17q21 and 13q13, respectively), LD was not assessed between the two genes.

Haplotypes were first estimated separately within each gene. No *BRCA1* haplotype was associated with RIF (global score test *p* = 0.066; Table S6). Among the *BRCA2* haplotypes (global *p* = 0.024), the A-A-A haplotype (rs144848–rs15869–rs1799943), which carries the rs1799943 A allele, was associated with lower odds of RIF relative to the A-A-G haplotype carrying the major allele at all three loci (AOR 0.551, 95% CI 0.376–0.807, *p* = 0.002, *q* = 0.012; Table S7). The rs15869 C allele occurred almost exclusively on haplotypes carrying the rs1799943 G allele, indicating that the nominal associations of rs15869-containing genotype combinations may partly reflect LD with rs1799943.

To summarize the joint distribution of alleles across both genes, seven-locus allele combinations were also estimated as an exploratory analysis (Table 4 and Table S8). Because *BRCA1* and *BRCA2* reside on different chromosomes and segregate independently, these combinations were inferred statistically by applying the expectation–maximization (EM) algorithm to the unphased seven-locus genotypes (Section 4.5); they represent multilocus allele combinations rather than physical haplotypes, and their frequencies reflect the joint distribution of genotypes at the two genes in the same participants. Relative to the combination carrying the major allele at all seven loci (G-T-G-C-A-A-G), the A-C-A-A-A-A-A combination was associated with lower odds of RIF (AOR 0.387, 95% CI 0.169–0.884, *p* = 0.025), whereas the A-C-A-A-A-A-G (AOR 3.289, 95% CI 1.247–8.676, *p* = 0.017) and A-C-A-A-A-C-G (AOR 3.792, 95% CI 1.328–10.825, *p* = 0.013) combinations were associated with higher odds (global score test *p* < 0.001); none of these associations remained significant after FDR correction (all *q* = 0.091). The A-C-A-A-A-A-A and A-C-A-A-A-A-G combinations differ only at rs1799943 and both carry the minor-allele *BRCA1* haplotype (A-C-A-A), whereas on the major-allele *BRCA1* background the rs1799943 A allele was not associated with RIF (G-T-G-C-A-A-A: AOR 0.891, 95% CI 0.525–1.512); this pattern mirrors the statistical interaction described above but does not indicate a biological mechanism or a synergistic effect between *BRCA1* and *BRCA2*. The gene-specific haplotype and allele-combination analyses were performed in the same participants as the single-locus analysis and therefore do not constitute independent validation of the rs1799943 association.

## 3. Discussion

### 3.1. Primary Single-Locus Analysis

In this case–control study of Korean women, we examined whether common polymorphisms in *BRCA1* and *BRCA2*, genes best known for their roles in hereditary cancer, are associated with recurrent implantation failure. The primary analysis consisted of single-locus tests of the seven SNPs under four genetic models with correction for multiple testing. In this analysis, the *BRCA2* rs1799943 A allele was associated with lower odds of RIF under the heterozygous and dominant models, and these associations remained significant after correction; no other SNP was associated with RIF after correction. All other analyses were exploratory and are discussed separately (Section 3.2). To our knowledge, this is the first study to examine *BRCA1/2* polymorphisms in relation to RIF; because no independent replication cohort was available, these findings should be regarded as hypothesis-generating.

BRCA1 and BRCA2 are essential for HR repair of DSBs, cell-cycle checkpoint regulation, and the maintenance of genomic stability [16,17], and BRCA-mediated DNA repair has been implicated in oocyte maintenance and ovarian function [23,30,31]. Because oocytes remain arrested in meiotic prophase for decades, they are particularly susceptible to the accumulation of DNA damage, and impaired DSB repair has been linked to oocyte loss and ovarian aging [23,24]. Successful implantation depends both on a euploid, developmentally competent embryo and on a receptive endometrium [5,10,11]. In the present cohort, RIF was defined on the basis of failed fresh transfers of cleavage-stage embryos selected by morphology alone, and embryo ploidy was not assessed. Because good morphology does not guarantee euploidy and embryo aneuploidy increases with maternal age [8,9], the associations observed here could reflect an influence of maternal *BRCA1/2* genotype on oocyte quality and embryonic chromosomal competence rather than, or in addition to, an influence on endometrial receptivity. Our data cannot distinguish between these possibilities, and conclusions regarding specific implantation mechanisms would be premature.

The allele-based analysis and the *BRCA2* haplotype analysis (Table S7) gave results concordant with the primary single-locus association of rs1799943. However, these analyses were derived from the same genotype data in the same participants and therefore do not constitute independent validation of the association, which will require replication in an independent cohort. rs1799943 (c.-26G>A) is located in the 5′ UTR of *BRCA2* (Figure 2). In reporter assays in MCF-7 breast cancer cells and HeLa cells, the A allele was associated with approximately twofold higher reporter expression than the G allele, and the G allele was predicted to stabilize a hairpin structure near the translation start site; in the same study, the heterozygous genotype was associated with a reduced risk of sporadic breast cancer [39]. It is therefore conceivable that the A allele enhances *BRCA2* expression and DSB repair capacity, but this has not been examined in oocytes, embryos, or endometrial cells, and the proposed mechanism remains a hypothesis. Allele-specific expression analyses, reporter assays in reproductive cell types, and DNA damage response profiling will be required to test it. Accordingly, we regard rs1799943 as a candidate variant associated with lower odds of RIF rather than as a protective biomarker.

### 3.2. Exploratory Analyses

The genotype-combination, interaction, haplotype, and allele-combination analyses were exploratory. They were performed in the same participants as the primary analysis, and their results, including the interaction and haplotype results in Tables S4 and S7, should be regarded as hypothesis-generating rather than as independent validation of the rs1799943 association. The interaction analysis suggested that the inverse association of rs1799943 was largely confined to carriers of the minor alleles of *BRCA1* rs12516, rs1799966, and rs8176318, which are in strong LD and together mark the minor-allele *BRCA1* haplotype (A-C-A-A). The seven-locus allele-combination analysis showed a corresponding pattern, with combinations carrying this *BRCA1* haplotype together with the rs1799943 G allele more frequent in RIF patients; however, because *BRCA1* and *BRCA2* segregate independently, these combinations are statistical constructs inferred by the EM algorithm and largely re-express the same cross-gene genotype associations. A statistical interaction on the multiplicative scale does not demonstrate biological interaction, the finding was not anticipated, and several genotype strata were small; neither the interaction nor the allele-combination results should therefore be interpreted as evidence of a biological mechanism or a synergistic effect between *BRCA1* and *BRCA2*. Although rs12516 and rs8176318 in the *BRCA1* 3′ UTR have been correlated with *BRCA1* mRNA expression [36] and rs1799966 is a missense variant (p.Ser1613Gly), whether these variants modify any effect of rs1799943 has not been examined, and the observed interaction pattern requires confirmation in independent cohorts. By contrast, the nominal associations of genotype combinations involving rs15869 were based on very few individuals, did not survive FDR correction, and were not supported by formal interaction tests. Because the rs15869 C allele occurred almost exclusively on haplotypes carrying the rs1799943 G allele, these patterns may partly reflect LD with rs1799943 rather than an independent effect of rs15869.

### 3.3. Limitations

Several limitations should be acknowledged. First, all participants were recruited from a single center and were of Korean ancestry, so the findings may not generalize to other populations, particularly because the allele frequencies of the studied SNPs differ across ethnic groups. Second, the controls were women with proven natural fertility rather than IVF patients who achieved pregnancy, and no such IVF-success group was available in our biobank. RIF patients and controls therefore differ in infertility status as well as in implantation outcome, and the observed associations may relate to infertility more broadly rather than specifically to implantation failure. Third, RIF was defined according to our institutional criteria on the basis of at least two failed fresh IVF–embryo transfer cycles with morphologically good-quality cleavage-stage embryos; this definition is less stringent than some contemporary definitions [5], embryo ploidy was not assessed by preimplantation genetic testing for aneuploidy (PGT-A), and maternal age at oocyte retrieval was approximated by age at enrollment. Fourth, recruitment spanned 2010 to 2023, during which IVF practice evolved. The same RIF criteria were applied throughout, and germline genotypes are not influenced by treatment era, so changes in practice would be expected mainly to cause non-differential misclassification of case status and to bias associations toward the null; however, enrollment dates were not available in the analysis dataset for a period-stratified analysis. Fifth, clinical variables were only available for subsets of participants, the timing of blood sampling relative to the menstrual cycle, fertility treatment, and pregnancy was not recorded, and sensitivity analyses in these subsets yielded estimates of varying magnitude, including an attenuated, non-significant estimate in the subset with TSH data. Sixth, although the primary single-locus analysis was corrected for multiple testing, the combination, interaction, haplotype, and allele-combination analyses were exploratory and were performed in the same participants, so they cannot validate the primary finding, and the numbers of individuals in some genotype categories were small. Finally, the SNPs were selected from WES data generated by our group; although the WES participants were not included in the present cohort, no independent replication cohort was available. Replication in larger, independent, and multi-ethnic cohorts, ideally including IVF patients with successful implantation as controls and embryo ploidy data, together with functional studies, is needed to confirm and extend these findings.

## 4. Materials and Methods

### 4.1. Study Approval and Study Population

This study complied with the code of ethics for human rights set out in the seventh revision (2013) of the 1975 Declaration of Helsinki. The study protocol was approved by the Institutional Review Board (IRB) of CHA Bundang Medical Center, Seongnam, Republic of Korea, on 23 February 2010 (reference no. PBC09-120), before the start of participant recruitment; following a change in the IRB numbering system, the protocol was re-registered under IRB File No. 2009-12-120 on 17 November 2015, and the approval has been maintained through continuing review. All methods were carried out in accordance with the approved guidelines. Written informed consent was obtained from all participants before enrollment and blood sample collection. Participants were recruited from the Department of Obstetrics and Gynecology (including its Fertility Center), CHA Bundang Medical Center, between March 2010 and November 2023.

A total of 458 Korean women were included: 128 patients with RIF and 330 controls. RIF was defined as the failure to achieve pregnancy following two or more completed fresh IVF–embryo transfer (IVF-ET) cycles in which one or two good-quality embryos were transferred per cycle; each transferred embryo had cleaved into more than 10 cells, and all transferred embryos were examined by an embryologist before transfer and judged to be of good quality [12]. Failure to achieve pregnancy was confirmed by a serum human chorionic gonadotropin (hCG) concentration of less than 5 mIU/mL 14 days after embryo transfer. Because RIF was defined on the basis of failed fresh cleavage-stage transfers, the embryos in the qualifying cycles were selected by morphology alone and were not screened by PGT-A; embryo ploidy was therefore unknown. Information on the cumulative number of embryos transferred per patient and on maternal age at each oocyte retrieval was not available in the analysis dataset, and age at enrollment was used as a proxy for maternal age.

Both partners of couples with RIF were evaluated, and patients with implantation failure attributable to anatomical, chromosomal, hormonal, infectious, autoimmune, or thrombotic causes were excluded. Anatomical abnormalities were evaluated by sonography, hysterosalpingography, hysteroscopy, computed tomography, and magnetic resonance imaging, and karyotyping was performed using standard protocols. Hormonal causes, including hyperprolactinemia, luteal insufficiency, and thyroid disease, were excluded by measuring prolactin, TSH, free thyroxine, follicle-stimulating hormone, luteinizing hormone, and progesterone in peripheral blood. Lupus anticoagulant and anticardiolipin antibodies were measured to exclude autoimmune causes such as lupus and antiphospholipid syndrome, and thrombophilia was evaluated by assessing protein C and protein S deficiencies and anti-β2-glycoprotein I antibodies. Semen analysis, karyotyping, and hormonal assays, including estradiol, testosterone, follicle-stimulating hormone, and luteinizing hormone, were performed for male partners. These diagnostic tests were performed in the clinical laboratory of CHA Bundang Medical Center using standard clinical assays. Of the 176 patients initially screened, 48 were excluded because of hypothyroidism, trisomy, intrauterine adhesions, other chromosomal disorders, or antiphospholipid syndrome, resulting in a study population of 128 patients. Smokers were excluded from both the RIF and control groups.

Controls were women recruited from the same department who had a history of regular menstrual cycles, a normal 46,XX karyotype, and at least one naturally conceived pregnancy. Women with proven natural fertility were selected as controls to represent women without impaired reproductive capacity, consistent with our previous studies of RIF [12]; IVF patients who achieved pregnancy were not available as an alternative control group. Clinical data and blood samples were collected in the same manner for both groups.

### 4.2. Biochemical, Hormonal, and Hematological Assessment

Blood samples were obtained at enrollment after an overnight fast of 8–10 h, and plasma and serum were separated by centrifugation at 1000× *g* for 15 min. Follicle-stimulating hormone, luteinizing hormone, and estradiol were measured using enzyme-linked immunoassay kits (Siemens, Munich, Germany), and TSH and prolactin were analyzed by radioimmunoassay (Beckman Coulter, Brea, CA, USA). Total cholesterol, uric acid, BUN, glucose, and creatinine were quantified by enzymatic colorimetry (Roche Diagnostics, Mannheim, Germany). Homocysteine and folate were measured by fluorescence polarization immunoassay (Abbott IMx analyzer; Abbott, Abbott Park, IL, USA) and competitive immunoassay (ACS 180 Plus; Bayer Diagnostics, Tarrytown, NY, USA), respectively. Complete blood counts were determined with an automated hematology analyzer (Sysmex XE-2100; Sysmex Corporation, Kobe, Japan). PT and aPTT were measured with an ACL TOP coagulation analyzer (Instrumentation Laboratory, Bedford, MA, USA). The timing of blood sampling relative to the menstrual cycle, fertility treatment, and, for controls, pregnancy was not systematically recorded. Not all laboratory tests were performed for every participant; the number of participants with available data for each variable is shown in Table 1.

### 4.3. Genomic DNA Extraction and SNP Selection

Genomic DNA was extracted from peripheral blood leukocytes using the G-DEX™ II Genomic DNA Extraction Kit (Intron Biotechnology, Seongnam, Republic of Korea; Cat. No. 17231) according to the manufacturer’s instructions.

Seven SNPs within *BRCA1* and *BRCA2* were selected on the basis of WES data generated by our group from two discovery sets: 35 women with primary ovarian insufficiency (POI) and 20 women with RIF, each compared with the same 40 healthy controls. Variant calling and annotation followed the discovery pipeline described previously [37]. Variants located within the *BRCA1* or *BRCA2* genomic regions that showed a significant allele-frequency difference between patients and controls (Fisher’s exact test, *p* < 0.05) in both the POI and the RIF WES datasets were retained. To ensure population relevance, candidate variants were cross-referenced with Korean reference datasets, including the Korean Genome Project 1K and 4K panels and the Korean Variant Archive (KOVA/KOGDB), and only SNPs with a minor allele frequency ≥0.05 in these panels were retained. POI was included as a discovery population because BRCA-mediated DSB repair has been implicated in ovarian aging and oocyte quality [23,25], which are also relevant to implantation competence. The WES participants were not included in the present genotyping cohort. The final panel comprised *BRCA1* rs12516 G>A, rs1799966 T>C, rs799917 G>A, and rs8176318 C>A, and *BRCA2* rs144848 A>C, rs15869 A>C, and rs1799943 G>A (Figure 2).

### 4.4. Genotyping

Genotyping was performed using TaqMan™ SNP Genotyping Assays (Applied Biosystems, Foster City, CA, USA; Cat. No. 4351379) on an RG-6000 real-time PCR system (Corbett Research, Sydney, Australia). Each 20 µL reaction contained genomic DNA, 10 µL of TaqMan™ Universal PCR Master Mix (Applied Biosystems; Cat. No. 4444965), and 0.5 µL of the allele-specific probe mix. Thermal cycling consisted of an initial denaturation at 95 °C for 10 min, followed by 40 cycles of 95 °C for 15 s and 60 °C for 1 min. To verify genotyping accuracy, 15% of samples were randomly selected and subjected to direct sequencing on an ABI 3730XL DNA Analyzer (Applied Biosystems); concordance between TaqMan and sequencing genotypes was 100%.

### 4.5. Statistical Analysis

Statistical analyses were performed using GraphPad Prism 9.0 (GraphPad Software, San Diego, CA, USA), MedCalc v20.2.18 (MedCalc Software Ltd., Ostend, Belgium), Python 3.13 (Python Software Foundation; https://www.python.org) with the SciPy (version 1.18.1) and statsmodels (version 0.15.0) packages, R 4.3.3 (R Foundation for Statistical Computing, Vienna, Austria) with the haplo.stats package (version 1.9.5.1), and Haploview version 4.2 (Broad Institute, Cambridge, MA, USA). Continuous variables are expressed as mean ± standard deviation and were compared between groups using Student’s *t*-test or, when Levene’s test indicated unequal variances, Welch’s *t*-test; normality was assessed with the Kolmogorov–Smirnov test. Clinical variables were analyzed using available data, and no imputation was performed. Categorical variables were compared using the chi-square test or Fisher’s exact test. Deviation from Hardy–Weinberg equilibrium was tested in each group using the chi-square test.

Single-locus associations between genotypes and RIF constituted the primary analysis and were estimated as odds ratios with 95% confidence intervals using logistic regression adjusted for age, under codominant (heterozygous and homozygous), dominant, and recessive models. Age was the only covariate in the primary models because germline genotypes are fixed at conception and cannot be influenced by clinical characteristics measured in adulthood, so that such characteristics do not act as conventional confounders of genotype–phenotype associations, and because several clinical variables were only available for subsets of participants. As sensitivity analyses, the models for rs1799943 were additionally adjusted for BMI or TSH in participants with available data, and participants with TSH > 4.5 µIU/mL were excluded. Multiplicity in the primary analysis (7 SNPs × 4 genetic models = 28 tests) was controlled using the Benjamini–Hochberg false discovery rate (FDR) [38]; Bonferroni-corrected *p* values were also examined, and associations with *q* < 0.05 were considered significant after correction. Allelic odds ratios were calculated with the chi-square test.

All other analyses were considered exploratory. In the genotype-combination analysis, each pairwise combination of genotypes was compared with the combination homozygous for the major allele at both loci using age-adjusted logistic regression, and FDR correction was applied across all 142 comparisons. Formal tests of SNP × SNP interaction were performed for all 21 SNP pairs by adding a multiplicative interaction term to age-adjusted logistic regression models including both main effects, with SNPs coded log-additively (0, 1, or 2 minor alleles) or dominantly; *p* values were obtained by likelihood ratio tests, and FDR correction was applied within each coding. Pairwise LD (D′) was estimated within each gene in all participants using Haploview. Haplotypes were estimated separately for *BRCA1* (four SNPs) and *BRCA2* (three SNPs) using the expectation–maximization (EM) algorithm. To describe the joint distribution of alleles across the two genes, the EM algorithm was also applied to the unphased seven-locus genotypes, treating the seven SNPs as a single multilocus unit under the assumption of Hardy–Weinberg equilibrium. Because *BRCA1* and *BRCA2* are located on different chromosomes and segregate independently, the phase inferred between the two genes has no physical meaning, and the resulting seven-locus combinations are statistical constructs rather than physical haplotypes. If genotypes at the two genes were independent, the frequency of each combination would be expected to approximate the product of the corresponding *BRCA1* and *BRCA2* haplotype frequencies; departures from this expectation therefore reflect cross-gene genotype associations in the same participants, which were tested formally by the interaction analysis described above. The seven-locus analysis was accordingly regarded as exploratory and descriptive and was not used to infer a biological interaction or synergistic effect between *BRCA1* and *BRCA2*. Associations of haplotypes and allele combinations with a frequency ≥1% were evaluated by haplotype-based logistic regression adjusted for age (haplo.glm), with the combination carrying the major allele at all loci as the reference, and global associations were assessed with the score test (haplo.score) with permutation [40]. Two-sided *p* values < 0.05 were considered nominally significant.

## 5. Conclusions

In this exploratory case–control study of Korean women, the *BRCA2* rs1799943 A allele was associated with lower odds of recurrent implantation failure, and this association remained significant after correction for multiple testing. Exploratory interaction, haplotype, and allele-combination analyses in the same participants were consistent with this association but do not constitute independent validation, and they do not provide evidence of a biological mechanism or a synergistic effect between *BRCA1* and *BRCA2*. Because embryo ploidy was unknown and the controls were naturally fertile women, these findings cannot distinguish effects on embryonic competence from effects on endometrial receptivity or on infertility more broadly. rs1799943 should be regarded as a candidate variant that warrants replication in independent cohorts and functional investigation, rather than as a biomarker for clinical risk stratification.

## Figures and Tables

**Table 1:** Clinical characteristics of patients with recurrent implantation failure (RIF) and control subjects.

Characteristic	Controls (n = 330)	RIF (n = 128)	*p*
Age (years)	33.44 ± 4.05 (330)	34.06 ± 3.16 (128)	0.112
BMI (kg/m^2^)	21.66 ± 3.37 (114)	21.39 ± 3.26 (126)	0.533
FSH (mIU/mL)	8.65 ± 4.58 (117)	8.69 ± 4.60 (91)	0.940
E2 (pg/mL)	30.48 ± 29.69 (109)	58.66 ± 134.35 (104)	0.039 ^a^
LH (mIU/mL)	3.96 ± 4.77 (109)	5.48 ± 2.76 (90)	0.008
TSH (µIU/mL)	1.58 ± 1.12 (130)	2.16 ± 1.38 (102)	<0.001
PT (s)	10.59 ± 1.49 (208)	11.32 ± 1.47 (123)	<0.001
aPTT (s)	29.32 ± 3.61 (210)	31.17 ± 4.00 (123)	<0.001
Homocysteine (µmol/L)	7.12 ± 2.75 (22)	6.73 ± 1.51 (64)	0.540 ^a^
Folate (ng/mL)	16.06 ± 12.35 (28)	15.27 ± 8.31 (44)	0.748
Glucose (mg/dL)	96.68 ± 18.81 (192)	96.17 ± 13.80 (110)	0.789 ^a^
BUN (mg/dL)	8.84 ± 2.78 (209)	10.48 ± 2.41 (121)	<0.001
Creatinine (mg/dL)	0.60 ± 0.15 (209)	0.75 ± 0.12 (121)	<0.001
Uric acid (mg/dL)	3.88 ± 0.97 (154)	4.06 ± 0.97 (88)	0.177
Total cholesterol (mg/dL)	230.62 ± 53.90 (185)	179.84 ± 31.71 (116)	<0.001 ^a^
WBC (10^3^/µL)	7.15 ± 2.46 (302)	5.81 ± 1.54 (123)	<0.001 ^a^
RBC (10^6^/µL)	4.12 ± 0.37 (302)	4.33 ± 0.34 (120)	<0.001
Hgb (g/dL)	12.39 ± 2.00 (304)	12.99 ± 0.96 (123)	0.002
Hct (%)	36.52 ± 3.12 (307)	38.65 ± 2.75 (120)	<0.001
MCV (fL)	88.17 ± 6.98 (302)	89.04 ± 5.30 (120)	0.217
MCH (pg)	29.90 ± 2.25 (302)	29.94 ± 1.56 (120)	0.835 ^a^
MCHC (g/dL)	33.68 ± 1.24 (302)	33.47 ± 1.08 (120)	0.094
RDW (%)	13.34 ± 1.26 (302)	12.89 ± 0.89 (120)	<0.001 ^a^
PLT (10^3^/µL)	237.00 ± 61.90 (308)	259.79 ± 63.50 (124)	0.001
PDW (fL)	12.46 ± 2.34 (144)	15.86 ± 18.18 (46)	0.213 ^a^
MPV (fL)	9.17 ± 4.88 (247)	8.78 ± 1.50 (113)	0.416
Seg (%)	69.91 ± 8.74 (201)	57.33 ± 8.75 (88)	<0.001
Lym (%)	21.65 ± 7.34 (201)	33.27 ± 6.70 (88)	<0.001
Mono (%)	5.36 ± 1.76 (201)	6.06 ± 3.40 (88)	0.023
Eo (%)	1.56 ± 1.32 (195)	2.34 ± 1.90 (85)	<0.001 ^a^
Baso (%)	0.35 ± 0.25 (188)	0.52 ± 0.32 (83)	<0.001

Data are presented as mean ± standard deviation (number of subjects with available data); clinical variables were not available for all participants, and comparisons were based on available data. *p* values were calculated by Student’s *t*-test or, when Levene’s test indicated unequal variances, by Welch’s *t*-test (^a^). BMI, body mass index; FSH, follicle-stimulating hormone; E2, estradiol; LH, luteinizing hormone; TSH, thyroid-stimulating hormone; PT, prothrombin time; aPTT, activated partial thromboplastin time; BUN, blood urea nitrogen; WBC, white blood cells; RBC, red blood cells; Hgb, hemoglobin; Hct, hematocrit; MCV, mean corpuscular volume; MCH, mean corpuscular hemoglobin; MCHC, mean corpuscular hemoglobin concentration; RDW, red cell distribution width; PLT, platelets; PDW, platelet distribution width; MPV, mean platelet volume; Seg, segmented neutrophils; Lym, lymphocytes; Mono, monocytes; Eo, eosinophils; Baso, basophils.

**Table 2:** Genotype frequencies of seven *BRCA1/2* polymorphisms in patients with recurrent implantation failure (RIF) and controls.

Genotype	Controls (n = 330)	RIF (n = 128)	COR (95% CI)	*p*	AOR (95% CI)	*p*	*q*
*BRCA1* rs12516 G>A							
GG	190 (57.6)	61 (47.7)	1.000 (reference)		1.000 (reference)		
GA	112 (33.9)	52 (40.6)	1.446 (0.934–2.240)	0.098	1.389 (0.893–2.161)	0.145	0.679
AA	28 (8.5)	15 (11.7)	1.669 (0.837–3.328)	0.146	1.589 (0.793–3.186)	0.192	0.679
Dominant (GG vs. GA + AA)			1.491 (0.989–2.246)	0.056	1.435 (0.948–2.171)	0.087	0.612
Recessive (GG + GA vs. AA)			1.432 (0.738–2.779)	0.289	1.393 (0.716–2.710)	0.328	0.679
HWE *p*	0.055	0.448					
*BRCA1 *rs1799966 T>C							
TT	176 (53.3)	64 (50.0)	1.000 (reference)		1.000 (reference)		
TC	125 (37.9)	51 (39.8)	1.122 (0.727–1.731)	0.603	1.082 (0.699–1.674)	0.725	0.752
CC	29 (8.8)	13 (10.2)	1.233 (0.604–2.518)	0.566	1.178 (0.575–2.417)	0.654	0.733
Dominant (TT vs. TC + CC)			1.143 (0.760–1.719)	0.522	1.104 (0.732–1.667)	0.637	0.733
Recessive (TT + TC vs. CC)			1.173 (0.589–2.336)	0.649	1.143 (0.573–2.280)	0.704	0.752
HWE *p*	0.319	0.551					
*BRCA1 *rs799917 G>A							
GG	189 (57.3)	66 (51.6)	1.000 (reference)		1.000 (reference)		
GA	115 (34.8)	50 (39.1)	1.245 (0.806–1.923)	0.323	1.230 (0.795–1.903)	0.352	0.679
AA	26 (7.9)	12 (9.4)	1.322 (0.631–2.768)	0.460	1.302 (0.621–2.731)	0.485	0.693
Dominant (GG vs. GA + AA)			1.259 (0.836–1.897)	0.270	1.242 (0.823–1.873)	0.301	0.679
Recessive (GG + GA vs. AA)			1.210 (0.591–2.477)	0.603	1.185 (0.578–2.431)	0.644	0.733
HWE *p*	0.156	0.575					
*BRCA1 *rs8176318 C>A							
CC	171 (51.8)	62 (48.4)	1.000 (reference)		1.000 (reference)		
CA	130 (39.4)	52 (40.6)	1.103 (0.715–1.702)	0.657	1.066 (0.689–1.650)	0.774	0.774
AA	29 (8.8)	14 (10.9)	1.331 (0.661–2.684)	0.423	1.278 (0.631–2.588)	0.495	0.693
Dominant (CC vs. CA + AA)			1.145 (0.761–1.722)	0.516	1.105 (0.732–1.669)	0.633	0.733
Recessive (CC + CA vs. AA)			1.275 (0.650–2.499)	0.480	1.242 (0.632–2.440)	0.529	0.705
HWE *p*	0.548	0.537					
*BRCA2 *rs144848 A>C							
AA	199 (60.3)	70 (54.7)	1.000 (reference)		1.000 (reference)		
AC	111 (33.6)	47 (36.7)	1.204 (0.778–1.863)	0.405	1.213 (0.783–1.879)	0.388	0.679
CC	20 (6.1)	11 (8.6)	1.564 (0.714–3.426)	0.264	1.584 (0.721–3.483)	0.252	0.679
Dominant (AA vs. AC + CC)			1.259 (0.834–1.900)	0.274	1.259 (0.833–1.902)	0.275	0.679
Recessive (AA + AC vs. CC)			1.457 (0.678–3.134)	0.335	1.402 (0.651–3.022)	0.388	0.679
HWE *p*	0.395	0.445					
*BRCA2 *rs15869 A>C							
AA	227 (68.8)	82 (64.1)	1.000 (reference)		1.000 (reference)		
AC	91 (27.6)	39 (30.5)	1.186 (0.755–1.865)	0.459	1.189 (0.756–1.870)	0.455	0.693
CC	12 (3.6)	7 (5.5)	1.615 (0.615–4.242)	0.331	1.555 (0.587–4.116)	0.374	0.679
Dominant (AA vs. AC + CC)			1.236 (0.805–1.900)	0.333	1.235 (0.803–1.900)	0.337	0.679
Recessive (AA + AC vs. CC)			1.533 (0.590–3.985)	0.381	1.493 (0.572–3.894)	0.413	0.680
HWE *p*	0.448	0.415					
*BRCA2 *rs1799943 G>A							
GG	102 (30.9)	65 (50.8)	1.000 (reference)		1.000 (reference)		
GA	169 (51.2)	47 (36.7)	0.436 (0.279–0.683)	<0.001 *	0.445 (0.284–0.698)	<0.001 *	0.006 *
AA	59 (17.9)	16 (12.5)	0.426 (0.226–0.802)	0.008 *	0.449 (0.237–0.850)	0.014 *	0.130
Dominant (GG vs. GA + AA)			0.434 (0.286–0.659)	<0.001 *	0.443 (0.291–0.674)	<0.001 *	0.004 *
Recessive (GG + GA vs. AA)			0.656 (0.362–1.189)	0.165	0.671 (0.370–1.218)	0.190	0.679
HWE *p*	0.446	0.114					

Data are presented as n (%). COR, crude odds ratio; AOR, adjusted odds ratio (adjusted for age); CI, confidence interval; HWE, Hardy–Weinberg equilibrium; *q*, Benjamini–Hochberg false discovery rate across the 28 age-adjusted tests (7 SNPs × 4 genetic models). * *p* < 0.05 or *q* < 0.05.

**Table 3:** Genotype combinations of *BRCA1/2* polymorphisms showing nominal associations with recurrent implantation failure (RIF).

Genotype Combination	Controls (n = 330)	RIF (n = 128)	AOR (95% CI)	*p*	*q*
***BRCA1*****rs12516 G>A** /***BRCA2*** **rs144848 A>C**					
GG/AA	108 (32.7)	32 (25.0)	1.000 (reference)		
AA/AC	7 (2.1)	7 (5.5)	3.347 (1.090–10.273)	0.035 *	0.380
***BRCA1*****rs12516 G>A**/***BRCA2*****rs15869 A>C**					
GG/AA	124 (37.6)	43 (33.6)	1.000 (reference)		
AA/AC †	4 (1.2)	7 (5.5)	5.210 (1.446–18.777)	0.012 *	0.261
***BRCA1*****rs12516 G>A**/***BRCA2*****rs1799943 G>A**					
GG/GG	70 (21.2)	27 (21.1)	1.000 (reference)		
GA/GG	26 (7.9)	29 (22.7)	2.698 (1.338–5.437)	0.006 *	0.261
AA/GG	6 (1.8)	9 (7.0)	3.770 (1.210–11.743)	0.022 *	0.261
***BRCA1*****rs1799966 T>C**/***BRCA2*****rs15869 A>C**					
TT/AA	118 (35.8)	46 (35.9)	1.000 (reference)		
CC/AC †	4 (1.2)	7 (5.5)	4.701 (1.304–16.951)	0.018 *	0.261
***BRCA1*****rs799917 G>A**/***BRCA2*****rs1799943 G>A**					
GG/GG	66 (20.0)	32 (25.0)	1.000 (reference)		
GG/GA	92 (27.9)	21 (16.4)	0.473 (0.251–0.894)	0.021 *	0.261
***BRCA1*****rs8176318 C>A**/***BRCA2*****rs15869 A>C**					
CC/AA	113 (34.2)	44 (34.4)	1.000 (reference)		
AA/AC †	4 (1.2)	7 (5.5)	4.618 (1.282–16.627)	0.019 *	0.261
***BRCA2*****rs144848 A>C**/***BRCA2*****rs15869 A>C**					
AA/AA	150 (45.5)	49 (38.3)	1.000 (reference)		
AC/CC †	2 (0.6)	6 (4.7)	8.885 (1.718–45.956)	0.009 *	0.261
***BRCA2*****rs144848 A>C**/***BRCA2*****rs1799943 G>A**					
AA/GG	47 (14.2)	31 (24.2)	1.000 (reference)		
AA/GA	99 (30.0)	26 (20.3)	0.391 (0.208–0.735)	0.004 *	0.250
AA/AA	53 (16.1)	13 (10.2)	0.369 (0.172–0.792)	0.011 *	0.261
AC/GA	69 (20.9)	20 (15.6)	0.449 (0.228–0.883)	0.020 *	0.261
***BRCA2*****rs15869 A>C**/***BRCA2*****rs1799943 G>A**					
AA/GG	50 (15.2)	34 (26.6)	1.000 (reference)		
AA/GA	118 (35.8)	32 (25.0)	0.407 (0.226–0.733)	0.003 *	0.250
AA/AA	59 (17.9)	16 (12.5)	0.429 (0.210–0.876)	0.020 *	0.261

Data are presented as n (%). Only combinations with *p* < 0.05 are shown, together with the reference combination (homozygous for the major allele at both loci) for each SNP pair; complete results for all 21 SNP pairs (142 comparisons) are provided in Table S3. AOR, adjusted odds ratio (adjusted for age), estimated by comparing each combination with the reference combination; CI, confidence interval; *q*, Benjamini–Hochberg false discovery rate across all 142 comparisons. † Fewer than five individuals in at least one group; estimates are imprecise. * *p* < 0.05.

**Table 4:** Seven-locus allele combinations of *BRCA1/2* polymorphisms showing nominal associations with recurrent implantation failure (RIF) in the exploratory allele-combination analysis.

Allele Combination	Controls (2n = 660)	RIF (2n = 256)	AOR (95% CI)	*p*	*q*
***BRCA1*** **rs12516–rs1799966–rs799917–rs8176318/** ***BRCA2*** **rs144848–rs15869–rs1799943**					
G-T-G-C-A-A-G	134 (20.2)	58 (22.5)	1.000 (reference)		
A-C-A-A-A-A-A	79 (11.9)	11 (4.3)	0.387 (0.169–0.884)	0.025 *	0.091
A-C-A-A-A-A-G	19 (2.9)	27 (10.6)	3.289 (1.247–8.676)	0.017 *	0.091
A-C-A-A-A-C-G	10 (1.5)	16 (6.1)	3.792 (1.328–10.825)	0.013 *	0.091

Data are presented as the estimated number of chromosomes (%), derived from expectation–maximization frequency estimates obtained from the unphased seven-locus genotypes in each group. Allele combinations are listed in the order *BRCA1* rs12516–rs1799966–rs799917–rs8176318/*BRCA2* rs144848–rs15869–rs1799943; because *BRCA1* and *BRCA2* are located on different chromosomes and segregate independently, these are statistically inferred multilocus allele combinations rather than physical haplotypes. These exploratory results describe the joint distribution of alleles across the two genes and do not indicate a biological mechanism or a synergistic effect between *BRCA1* and *BRCA2*. Only combinations with *p* < 0.05 are shown together with the reference combination (major allele at all loci); complete results for all combinations with a frequency ≥1% are provided in Table S8. AOR, adjusted odds ratio per copy (adjusted for age) from haplotype-based logistic regression; CI, confidence interval; *q*, Benjamini–Hochberg false discovery rate across all estimable combinations. Global score test *p* < 0.001. * *p* < 0.05.

## Data Availability

The data presented in this study are available on request from the corresponding author due to ethical restrictions on sharing individual genotype and clinical data.

## Supplementary Materials

The following supporting information can be downloaded at: https://www.mdpi.com/article/10.3390/ijms272110032/s1.
