# Supplementary material for: BRCA1/2 Polymorphisms and the Risk of Recurrent Implantation Failure in Korean Women
Source: Int J Mol Sci. 2026 Nov 9;27(21):10032. doi: 10.3390/ijms272110032 (PMC13764409; doi:10.3390/ijms272110032)
Supplement: Supplementary file 1 [file ijms-27-10032-s001.zip › ijms-4628074-supplementary.pdf]

Supplementary Materials

BRCA1/2 Polymorphisms and the Risk of Recurrent Implantation Failure in Korean Women

Hyeon Woo Park, Da Hwan Kim, Ji Hyang Kim, So Yeon Shin, Ji Eun Ko and Nam Keun Kim

Abbreviations used throughout: RIF, recurrent implantation failure; AOR, adjusted odds ratio (adjusted for age); CI, confidence interval; OR, odds ratio; *q*, Benjamini–Hochberg false discovery rate; NE, not estimable. * $p < 0.05$ (or $q < 0.05$ in *q* columns). Tables S3–S8 present exploratory analyses performed in the same participants as the primary single-locus analysis (Table 2 of the main text); they do not constitute independent validation of the primary findings.

Table S1. Allele frequencies of the seven *BRCA1/2* polymorphisms in patients with RIF and controls.

SNP	Controls (ref/var alleles)	RIF (ref/var alleles)	MAF controls	MAF RIF	OR (95 % CI)	*p*	*q*
BRCA1 rs12516 G>A	492 / 168	174 / 82	0.255	0.320	1.380 (1.007–1.892)	0.045 *	0.157
BRCA1 rs1799966 T>C	477 / 183	179 / 77	0.277	0.301	1.121 (0.817–1.539)	0.479	0.479
BRCA1 rs799917 G>A	493 / 167	182 / 74	0.253	0.289	1.200 (0.870–1.657)	0.266	0.373
BRCA1 rs8176318 C>A	472 / 188	176 / 80	0.285	0.312	1.141 (0.834–1.562)	0.409	0.477
BRCA2 rs144848 A>C	509 / 151	187 / 69	0.229	0.270	1.244 (0.894–1.731)	0.195	0.373
BRCA2 rs15869 A>C	545 / 115	203 / 53	0.174	0.207	1.237 (0.861–1.779)	0.250	0.373
BRCA2 rs1799943 G>A	373 / 287	177 / 79	0.435	0.309	0.580 (0.427–0.788)	< 0.001 *	0.003 *

Allele counts are shown as reference/variant alleles (controls, 2n = 660; RIF, 2n = 256). MAF, variant (minor) allele frequency; OR, allelic odds ratio for the variant allele (chi-square test); *q*, false discovery rate across the seven SNPs.

Table S2. Sensitivity analyses of the association between *BRCA2* rs1799943 and RIF.

Comparison	n (controls/RIF)	AOR (95 % CI)	*p*
**Age (all participants)** (controls, 330; RIF, 128)			
GA vs. GG	271 / 112	0.445 (0.284–0.698)	< 0.001 *
AA vs. GG	161 / 81	0.449 (0.237–0.850)	0.014 *
GA + AA vs. GG	330 / 128	0.443 (0.291–0.674)	< 0.001 *
**Age (participants with BMI data)** (controls, 114; RIF, 126)			
GA vs. GG	90 / 110	0.263 (0.142–0.486)	< 0.001 *
AA vs. GG	47 / 80	0.270 (0.119–0.612)	0.002 *
GA + AA vs. GG	114 / 126	0.262 (0.146–0.470)	< 0.001 *
**Age + BMI** (controls, 114; RIF, 126)			
GA vs. GG	90 / 110	0.258 (0.139–0.479)	< 0.001 *
AA vs. GG	47 / 80	0.269 (0.119–0.610)	0.002 *
GA + AA vs. GG	114 / 126	0.258 (0.143–0.463)	< 0.001 *
**Age (participants with TSH data)** (controls, 130; RIF, 102)			
GA vs. GG	108 / 89	0.682 (0.383–1.213)	0.192
AA vs. GG	73 / 65	0.628 (0.282–1.395)	0.253
GA + AA vs. GG	130 / 102	0.665 (0.390–1.133)	0.133
**Age + TSH** (controls, 130; RIF, 102)			
GA vs. GG	108 / 89	0.742 (0.408–1.350)	0.329
AA vs. GG	73 / 65	0.656 (0.291–1.477)	0.309
GA + AA vs. GG	130 / 102	0.712 (0.412–1.231)	0.224
**Age (excluding participants with TSH > 4.5 μIU/mL)** (controls, 327; RIF, 123)			
GA vs. GG	269 / 107	0.473 (0.299–0.746)	0.001 *
AA vs. GG	158 / 76	0.487 (0.256–0.929)	0.029 *
GA + AA vs. GG	327 / 123	0.473 (0.309–0.724)	< 0.001 *

The first model is the primary analysis (age-adjusted, all participants). For BMI and TSH, the age-adjusted estimate in the same subset of participants with available data is shown for comparison with the estimate additionally adjusted for the covariate. TSH values > 4.5 μIU/mL were observed in five patients with RIF and three controls. BMI, body mass index; TSH, thyroid-stimulating hormone.

Table S3. Complete results of the pairwise genotype-combination analysis of the seven *BRCA1/2* polymorphisms.

Genotype combination	Controls (n = 330)	RIF (n = 128)	AOR (95 % CI)	*p*	*q*
***BRCA1* rs12516 G>A / *BRCA1* rs1799966 T>C**					

Genotype combination	Controls (n = 330)	RIF (n = 128)	AOR (95 % CI)	*p*	*q*
GG/TT	173 (52.4)	61 (47.7)	1.000 (reference)		
GG/TC	15 (4.5)	0 (0.0)	NE		
GG/CC	2 (0.6)	0 (0.0)	NE		
GA/TT †	2 (0.6)	2 (1.6)	2.528 (0.344–18.556)	0.362	0.779
GA/TC	109 (33.0)	50 (39.1)	1.253 (0.800–1.962)	0.325	0.756
GA/CC	1 (0.3)	0 (0.0)	NE		
AA/TT †	1 (0.3)	1 (0.8)	2.445 (0.147–40.654)	0.533	0.846
AA/TC †	1 (0.3)	1 (0.8)	3.023 (0.186–49.206)	0.437	0.795
AA/CC	26 (7.9)	13 (10.2)	1.359 (0.654–2.826)	0.411	0.794
***BRCA1* rs12516 G>A / *BRCA1* rs799917 G>A**					
GG/GG	165 (50.0)	60 (46.9)	1.000 (reference)		
GG/GA	23 (7.0)	0 (0.0)	NE		
GG/AA †	2 (0.6)	1 (0.8)	1.409 (0.124–15.962)	0.782	0.895
GA/GG †	19 (5.8)	3 (2.3)	0.407 (0.115–1.448)	0.165	0.583
GA/GA	90 (27.3)	49 (38.3)	1.448 (0.914–2.294)	0.115	0.534
GA/AA	3 (0.9)	0 (0.0)	NE		
AA/GG †	5 (1.5)	3 (2.3)	1.565 (0.361–6.780)	0.549	0.846
AA/GA †	2 (0.6)	1 (0.8)	1.433 (0.127–16.128)	0.771	0.895
AA/AA	21 (6.4)	11 (8.6)	1.392 (0.630–3.074)	0.414	0.794
***BRCA1* rs12516 G>A / *BRCA1* rs8176318 C>A**					
GG/CC	168 (50.9)	61 (47.7)	1.000 (reference)		
GG/CA	19 (5.8)	0 (0.0)	NE		
GG/AA	3 (0.9)	0 (0.0)	NE		
GA/CC †	3 (0.9)	1 (0.8)	0.870 (0.088–8.556)	0.905	0.924
GA/CA	109 (33.0)	51 (39.8)	1.240 (0.793–1.940)	0.346	0.779
GA/AA	0 (0.0)	0 (0.0)	NE		
AA/CC	0 (0.0)	0 (0.0)	NE		
AA/CA †	2 (0.6)	1 (0.8)	1.464 (0.130–16.482)	0.758	0.895
AA/AA	26 (7.9)	14 (10.9)	1.410 (0.687–2.895)	0.348	0.779
***BRCA1* rs12516 G>A / *BRCA2* rs144848 A>C**					
GG/AA	108 (32.7)	32 (25.0)	1.000 (reference)		
GG/AC	70 (21.2)	23 (18.0)	1.113 (0.601–2.061)	0.734	0.895
GG/CC	12 (3.6)	6 (4.7)	1.718 (0.590–5.007)	0.321	0.756
GA/AA	74 (22.4)	31 (24.2)	1.425 (0.797–2.548)	0.232	0.633
GA/AC	34 (10.3)	17 (13.3)	1.617 (0.795–3.292)	0.185	0.583
GA/CC †	4 (1.2)	4 (3.1)	3.440 (0.810–14.617)	0.094	0.495
AA/AA	17 (5.2)	7 (5.5)	1.448 (0.546–3.844)	0.457	0.811
AA/AC	7 (2.1)	7 (5.5)	3.347 (1.090–10.273)	0.035 *	0.380
AA/CC †	4 (1.2)	1 (0.8)	0.853 (0.091–7.997)	0.889	0.924
***BRCA1* rs12516 G>A / *BRCA2* rs15869 A>C**					
GG/AA	124 (37.6)	43 (33.6)	1.000 (reference)		
GG/AC	61 (18.5)	14 (10.9)	0.651 (0.330–1.283)	0.215	0.633
GG/CC †	5 (1.5)	4 (3.1)	2.053 (0.514–8.204)	0.309	0.744
GA/AA	80 (24.2)	32 (25.0)	1.094 (0.635–1.888)	0.745	0.895
GA/AC	26 (7.9)	18 (14.1)	1.871 (0.927–3.780)	0.081	0.476
GA/CC †	6 (1.8)	2 (1.6)	0.904 (0.174–4.701)	0.905	0.924
AA/AA	23 (7.0)	7 (5.5)	0.753 (0.296–1.915)	0.551	0.846
AA/AC †	4 (1.2)	7 (5.5)	5.210 (1.446–18.777)	0.012 *	0.261
AA/CC †	1 (0.3)	1 (0.8)	2.594 (0.151–44.516)	0.511	0.846
***BRCA1* rs12516 G>A / *BRCA2* rs1799943 G>A**					
GG/GG	70 (21.2)	27 (21.1)	1.000 (reference)		
GG/GA	89 (27.0)	21 (16.4)	0.620 (0.323–1.189)	0.150	0.547
GG/AA	31 (9.4)	13 (10.2)	1.230 (0.548–2.760)	0.616	0.886
GA/GG	26 (7.9)	29 (22.7)	2.698 (1.338–5.437)	0.006 *	0.261
GA/GA	69 (20.9)	20 (15.6)	0.746 (0.382–1.455)	0.390	0.794
GA/AA †	17 (5.2)	3 (2.3)	0.448 (0.121–1.660)	0.230	0.633
AA/GG	6 (1.8)	9 (7.0)	3.770 (1.210–11.743)	0.022 *	0.261
AA/GA	11 (3.3)	6 (4.7)	1.439 (0.482–4.298)	0.515	0.846
AA/AA	11 (3.3)	0 (0.0)	NE		
***BRCA1* rs1799966 T>C / *BRCA1* rs799917 G>A**					
TT/GG	162 (49.1)	62 (48.4)	1.000 (reference)		

Genotype combination	Controls (n = 330)	RIF (n = 128)	AOR (95 % CI)	*p*	*q*
TT/GA †	12 (3.6)	1 (0.8)	0.234 (0.030–1.849)	0.168	0.583
TT/AA †	2 (0.6)	1 (0.8)	1.343 (0.119–15.222)	0.812	0.899
TC/GG †	20 (6.1)	2 (1.6)	0.243 (0.054–1.086)	0.064	0.462
TC/GA	101 (30.6)	49 (38.3)	1.234 (0.785–1.940)	0.362	0.779
TC/AA	4 (1.2)	0 (0.0)	NE		
CC/GG †	7 (2.1)	2 (1.6)	0.735 (0.148–3.637)	0.706	0.895
CC/GA	2 (0.6)	0 (0.0)	NE		
CC/AA	20 (6.1)	11 (8.6)	1.386 (0.625–3.076)	0.422	0.794
***BRCA1* rs1799966 T>C / *BRCA1* rs8176318 C>A**					
TT/CC	169 (51.2)	62 (48.4)	1.000 (reference)		
TT/CA †	5 (1.5)	1 (0.8)	0.521 (0.060–4.562)	0.556	0.846
TT/AA †	2 (0.6)	1 (0.8)	1.306 (0.115–14.875)	0.830	0.900
TC/CC	1 (0.3)	0 (0.0)	NE		
TC/CA	124 (37.6)	51 (39.8)	1.085 (0.699–1.686)	0.716	0.895
TC/AA	0 (0.0)	0 (0.0)	NE		
CC/CC	1 (0.3)	0 (0.0)	NE		
CC/CA	1 (0.3)	0 (0.0)	NE		
CC/AA	27 (8.2)	13 (10.2)	1.267 (0.612–2.621)	0.524	0.846
***BRCA1* rs1799966 T>C / *BRCA2* rs144848 A>C**					
TT/AA	101 (30.6)	34 (26.6)	1.000 (reference)		
TT/AC	63 (19.1)	24 (18.8)	1.134 (0.615–2.094)	0.687	0.895
TT/CC	12 (3.6)	6 (4.7)	1.460 (0.504–4.227)	0.486	0.831
TC/AA	81 (24.5)	30 (23.4)	1.098 (0.618–1.951)	0.751	0.895
TC/AC	40 (12.1)	17 (13.3)	1.208 (0.604–2.414)	0.593	0.877
TC/CC †	4 (1.2)	4 (3.1)	2.948 (0.697–12.473)	0.142	0.544
CC/AA	17 (5.2)	6 (4.7)	1.066 (0.387–2.939)	0.901	0.924
CC/AC	8 (2.4)	6 (4.7)	2.166 (0.699–6.712)	0.181	0.583
CC/CC †	4 (1.2)	1 (0.8)	0.716 (0.077–6.684)	0.769	0.895
***BRCA1* rs1799966 T>C / *BRCA2* rs15869 A>C**					
TT/AA	118 (35.8)	46 (35.9)	1.000 (reference)		
TT/AC	54 (16.4)	14 (10.9)	0.652 (0.329–1.289)	0.219	0.633
TT/CC †	4 (1.2)	4 (3.1)	2.228 (0.518–9.595)	0.282	0.690
TC/AA	85 (25.8)	31 (24.2)	0.888 (0.517–1.525)	0.667	0.895
TC/AC	33 (10.0)	18 (14.1)	1.318 (0.670–2.594)	0.423	0.794
TC/CC †	7 (2.1)	2 (1.6)	0.702 (0.139–3.546)	0.669	0.895
CC/AA	24 (7.3)	5 (3.9)	0.457 (0.162–1.290)	0.139	0.544
CC/AC †	4 (1.2)	7 (5.5)	4.701 (1.304–16.951)	0.018 *	0.261
CC/CC †	1 (0.3)	1 (0.8)	2.299 (0.133–39.891)	0.567	0.848
***BRCA1* rs1799966 T>C / *BRCA2* rs1799943 G>A**					
TT/GG	64 (19.4)	28 (21.9)	1.000 (reference)		
TT/GA	83 (25.2)	23 (18.0)	0.640 (0.337–1.218)	0.174	0.583
TT/AA	29 (8.8)	13 (10.2)	1.189 (0.525–2.695)	0.678	0.895
TC/GG	32 (9.7)	29 (22.7)	1.928 (0.976–3.806)	0.059	0.462
TC/GA	74 (22.4)	19 (14.8)	0.586 (0.299–1.149)	0.120	0.534
TC/AA †	19 (5.8)	3 (2.3)	0.357 (0.097–1.310)	0.120	0.534
CC/GG	6 (1.8)	8 (6.2)	3.084 (0.966–9.847)	0.057	0.462
CC/GA	12 (3.6)	5 (3.9)	0.959 (0.307–2.998)	0.943	0.949
CC/AA	11 (3.3)	0 (0.0)	NE		
***BRCA1* rs799917 G>A / *BRCA1* rs8176318 C>A**					
GG/CC	160 (48.5)	61 (47.7)	1.000 (reference)		
GG/CA †	21 (6.4)	2 (1.6)	0.238 (0.054–1.055)	0.059	0.462
GG/AA †	8 (2.4)	3 (2.3)	0.973 (0.249–3.794)	0.968	0.968
GA/CC	9 (2.7)	0 (0.0)	NE		
GA/CA	105 (31.8)	50 (39.1)	1.218 (0.776–1.910)	0.391	0.794
GA/AA	1 (0.3)	0 (0.0)	NE		
AA/CC †	2 (0.6)	1 (0.8)	1.339 (0.119–15.126)	0.813	0.899
AA/CA	4 (1.2)	0 (0.0)	NE		
AA/AA	20 (6.1)	11 (8.6)	1.407 (0.634–3.125)	0.401	0.794
***BRCA1* rs799917 G>A / *BRCA2* rs144848 A>C**					
GG/AA	116 (35.2)	34 (26.6)	1.000 (reference)		
GG/AC	61 (18.5)	25 (19.5)	1.392 (0.762–2.544)	0.282	0.690

Genotype combination	Controls (n = 330)	RIF (n = 128)	AOR (95 % CI)	*p*	*q*
GG/CC	12 (3.6)	7 (5.5)	2.011 (0.729–5.547)	0.177	0.583
GA/AA	66 (20.0)	30 (23.4)	1.552 (0.870–2.766)	0.136	0.544
GA/AC	45 (13.6)	17 (13.3)	1.287 (0.653–2.536)	0.467	0.815
GA/CC †	4 (1.2)	3 (2.3)	2.584 (0.550–12.145)	0.229	0.633
AA/AA	17 (5.2)	6 (4.7)	1.235 (0.449–3.391)	0.683	0.895
AA/AC	5 (1.5)	5 (3.9)	3.392 (0.926–12.423)	0.065	0.462
AA/CC †	4 (1.2)	1 (0.8)	0.856 (0.092–7.973)	0.892	0.924
***BRCA1* rs799917 G>A / *BRCA2* rs15869 A>C**					
GG/AA	129 (39.1)	46 (35.9)	1.000 (reference)		
GG/AC	54 (16.4)	16 (12.5)	0.819 (0.426–1.575)	0.550	0.846
GG/CC †	6 (1.8)	4 (3.1)	1.705 (0.452–6.427)	0.430	0.794
GA/AA	79 (23.9)	31 (24.2)	1.066 (0.622–1.826)	0.816	0.899
GA/AC	32 (9.7)	17 (13.3)	1.490 (0.752–2.951)	0.253	0.665
GA/CC †	4 (1.2)	2 (1.6)	1.337 (0.235–7.613)	0.743	0.895
AA/AA	19 (5.8)	5 (3.9)	0.662 (0.231–1.902)	0.444	0.798
AA/AC	5 (1.5)	6 (4.7)	3.419 (0.993–11.772)	0.051	0.462
AA/CC †	2 (0.6)	1 (0.8)	1.425 (0.123–16.507)	0.777	0.895
***BRCA1* rs799917 G>A / *BRCA2* rs1799943 G>A**					
GG/GG	66 (20.0)	32 (25.0)	1.000 (reference)		
GG/GA	92 (27.9)	21 (16.4)	0.473 (0.251–0.894)	0.021 *	0.261
GG/AA	31 (9.4)	13 (10.2)	0.944 (0.429–2.079)	0.886	0.924
GA/GG	28 (8.5)	27 (21.1)	1.916 (0.967–3.796)	0.062	0.462
GA/GA	68 (20.6)	20 (15.6)	0.616 (0.320–1.187)	0.148	0.547
GA/AA †	19 (5.8)	3 (2.3)	0.330 (0.091–1.199)	0.092	0.495
AA/GG	8 (2.4)	6 (4.7)	1.596 (0.507–5.032)	0.424	0.794
AA/GA	9 (2.7)	6 (4.7)	1.395 (0.456–4.269)	0.560	0.846
AA/AA	9 (2.7)	0 (0.0)	NE		
***BRCA1* rs8176318 C>A / *BRCA2* rs144848 A>C**					
CC/AA	96 (29.1)	32 (25.0)	1.000 (reference)		
CC/AC	64 (19.4)	24 (18.8)	1.123 (0.605–2.084)	0.712	0.895
CC/CC	11 (3.3)	6 (4.7)	1.672 (0.565–4.947)	0.353	0.779
CA/AA	84 (25.5)	31 (24.2)	1.113 (0.624–1.984)	0.718	0.895
CA/AC	41 (12.4)	17 (13.3)	1.200 (0.598–2.409)	0.608	0.886
CA/CC †	5 (1.5)	4 (3.1)	2.448 (0.616–9.727)	0.204	0.628
AA/AA	19 (5.8)	7 (5.5)	1.137 (0.435–2.975)	0.793	0.899
AA/AC	6 (1.8)	6 (4.7)	2.971 (0.891–9.905)	0.076	0.471
AA/CC †	4 (1.2)	1 (0.8)	0.761 (0.081–7.136)	0.811	0.899
***BRCA1* rs8176318 C>A / *BRCA2* rs15869 A>C**					
CC/AA	113 (34.2)	44 (34.4)	1.000 (reference)		
CC/AC	54 (16.4)	14 (10.9)	0.656 (0.331–1.302)	0.228	0.633
CC/CC †	4 (1.2)	4 (3.1)	2.291 (0.536–9.791)	0.263	0.667
CA/AA	90 (27.3)	32 (25.0)	0.872 (0.508–1.495)	0.618	0.886
CA/AC	33 (10.0)	18 (14.1)	1.329 (0.674–2.621)	0.411	0.794
CA/CC †	7 (2.1)	2 (1.6)	0.707 (0.140–3.560)	0.674	0.895
AA/AA	24 (7.3)	6 (4.7)	0.557 (0.209–1.479)	0.240	0.642
AA/AC †	4 (1.2)	7 (5.5)	4.618 (1.282–16.627)	0.019 *	0.261
AA/CC †	1 (0.3)	1 (0.8)	2.342 (0.138–39.755)	0.556	0.846
***BRCA1* rs8176318 C>A / *BRCA2* rs1799943 G>A**					
CC/GG	61 (18.5)	27 (21.1)	1.000 (reference)		
CC/GA	82 (24.8)	22 (17.2)	0.610 (0.317–1.174)	0.139	0.544
CC/AA	28 (8.5)	13 (10.2)	1.188 (0.522–2.704)	0.682	0.895
CA/GG	33 (10.0)	29 (22.7)	1.856 (0.937–3.677)	0.076	0.471
CA/GA	77 (23.3)	20 (15.6)	0.585 (0.300–1.143)	0.117	0.534
CA/AA †	20 (6.1)	3 (2.3)	0.336 (0.092–1.232)	0.100	0.507
AA/GG	8 (2.4)	9 (7.0)	2.547 (0.875–7.413)	0.086	0.491
AA/GA	10 (3.0)	5 (3.9)	1.136 (0.353–3.659)	0.830	0.900
AA/AA	11 (3.3)	0 (0.0)	NE		
***BRCA2* rs144848 A>C / *BRCA2* rs15869 A>C**					
AA/AA	150 (45.5)	49 (38.3)	1.000 (reference)		
AA/AC	40 (12.1)	21 (16.4)	1.616 (0.870–3.003)	0.129	0.544
AA/CC	9 (2.7)	0 (0.0)	NE		
AC/AA	65 (19.7)	26 (20.3)	1.229 (0.702–2.149)	0.471	0.815
AC/AC	44 (13.3)	15 (11.7)	1.056 (0.540–2.064)	0.874	0.924

Genotype combination	Controls (n = 330)	RIF (n = 128)	AOR (95 % CI)	*p*	*q*
AC/CC †	2 (0.6)	6 (4.7)	8.885 (1.718–45.956)	0.009 *	0.261
CC/AA	12 (3.6)	7 (5.5)	1.773 (0.658–4.775)	0.257	0.665
CC/AC †	7 (2.1)	3 (2.3)	1.312 (0.326–5.282)	0.702	0.895
CC/CC †	1 (0.3)	1 (0.8)	3.072 (0.189–50.064)	0.431	0.794
***BRCA2* rs144848 A>C / *BRCA2* rs1799943 G>A**					
AA/GG	47 (14.2)	31 (24.2)	1.000 (reference)		
AA/GA	99 (30.0)	26 (20.3)	0.391 (0.208–0.735)	0.004 *	0.250
AA/AA	53 (16.1)	13 (10.2)	0.369 (0.172–0.792)	0.011 *	0.261
AC/GG	40 (12.1)	26 (20.3)	1.034 (0.523–2.044)	0.923	0.937
AC/GA	69 (20.9)	20 (15.6)	0.449 (0.228–0.883)	0.020 *	0.261
AC/AA †	2 (0.6)	1 (0.8)	0.793 (0.067–9.363)	0.854	0.919
CC/GG	15 (4.5)	8 (6.2)	0.810 (0.306–2.139)	0.670	0.895
CC/GA †	1 (0.3)	1 (0.8)	1.521 (0.091–25.302)	0.770	0.895
CC/AA †	4 (1.2)	2 (1.6)	0.758 (0.131–4.393)	0.757	0.895
***BRCA2* rs15869 A>C / *BRCA2* rs1799943 G>A**					
AA/GG	50 (15.2)	34 (26.6)	1.000 (reference)		
AA/GA	118 (35.8)	32 (25.0)	0.407 (0.226–0.733)	0.003 *	0.250
AA/AA	59 (17.9)	16 (12.5)	0.429 (0.210–0.876)	0.020 *	0.261
AC/GG	44 (13.3)	24 (18.8)	0.808 (0.416–1.572)	0.531	0.846
AC/GA	47 (14.2)	15 (11.7)	0.512 (0.245–1.071)	0.076	0.471
AC/AA	0 (0.0)	0 (0.0)	NE		
CC/GG	8 (2.4)	7 (5.5)	1.284 (0.403–4.091)	0.672	0.895
CC/GA	4 (1.2)	0 (0.0)	NE		
CC/AA	0 (0.0)	0 (0.0)	NE		

Data are presented as n (%). For each SNP pair, each genotype combination was compared with the reference combination (homozygous for the major allele at both loci) by age-adjusted logistic regression. *q*, false discovery rate across all 142 estimable comparisons. † Fewer than five individuals in at least one group; estimates are imprecise. NE, not estimable because no individuals carried the combination in one group.

Table S4. Formal tests of SNP × SNP interaction on the multiplicative scale.

SNP pair	Ratio of ORs (95 % CI)	*p* (LRT)	*q*
**Log-additive coding**			
BRCA1 rs12516 × *BRCA1* rs1799966	1.073 (0.669–1.721)	0.771	0.900
BRCA1 rs12516 × *BRCA1* rs799917	1.177 (0.761–1.821)	0.463	0.799
BRCA1 rs12516 × *BRCA1* rs8176318	1.109 (0.691–1.781)	0.668	0.826
BRCA1 rs12516 × *BRCA2* rs144848	1.006 (0.638–1.586)	0.979	0.979
BRCA1 rs12516 × *BRCA2* rs15869	1.713 (0.986–2.976)	0.051	0.154
BRCA1 rs12516 × *BRCA2* rs1799943	0.401 (0.241–0.667)	< 0.001 *	0.003 *
BRCA1 rs1799966 × *BRCA1* rs799917	1.227 (0.788–1.912)	0.365	0.799
BRCA1 rs1799966 × *BRCA1* rs8176318	1.108 (0.693–1.770)	0.669	0.826
BRCA1 rs1799966 × *BRCA2* rs144848	1.034 (0.652–1.640)	0.888	0.975
BRCA1 rs1799966 × *BRCA2* rs15869	1.780 (1.019–3.111)	0.039 *	0.154
BRCA1 rs1799966 × *BRCA2* rs1799943	0.405 (0.241–0.678)	< 0.001 *	0.003 *
BRCA1 rs799917 × *BRCA1* rs8176318	1.202 (0.770–1.876)	0.419	0.799
BRCA1 rs799917 × *BRCA2* rs144848	0.854 (0.536–1.360)	0.505	0.799
BRCA1 rs799917 × *BRCA2* rs15869	1.403 (0.831–2.369)	0.202	0.531
BRCA1 rs799917 × *BRCA2* rs1799943	0.553 (0.337–0.907)	0.015 *	0.079
BRCA1 rs8176318 × *BRCA2* rs144848	1.021 (0.644–1.620)	0.929	0.975
BRCA1 rs8176318 × *BRCA2* rs15869	1.731 (0.992–3.021)	0.049 *	0.154
BRCA1 rs8176318 × *BRCA2* rs1799943	0.434 (0.263–0.718)	< 0.001 *	0.004 *
BRCA2 rs144848 × *BRCA2* rs15869	1.152 (0.662–2.004)	0.616	0.826
BRCA2 rs144848 × *BRCA2* rs1799943	1.163 (0.727–1.860)	0.532	0.799
BRCA2 rs15869 × *BRCA2* rs1799943	0.743 (0.366–1.510)	0.407	0.799
**Dominant coding**			
BRCA1 rs12516 × *BRCA1* rs1799966	NE ‡		
BRCA1 rs12516 × *BRCA1* rs799917	19.040 (2.041–177.632)	< 0.001 *	0.014 *
BRCA1 rs12516 × *BRCA1* rs8176318	NE ‡		
BRCA1 rs12516 × *BRCA2* rs144848	1.199 (0.522–2.753)	0.670	0.743
BRCA1 rs12516 × *BRCA2* rs15869	2.650 (1.098–6.398)	0.029 *	0.074
BRCA1 rs12516 × *BRCA2* rs1799943	0.309 (0.131–0.728)	0.007 *	0.050
BRCA1 rs1799966 × *BRCA1* rs799917	8.349 (1.287–54.156)	0.012 *	0.050
BRCA1 rs1799966 × *BRCA1* rs8176318	NE ‡		

SNP pair	Ratio of ORs (95 % CI)	p (LRT)	q
BRCA1 rs1799966 × *BRCA2* rs144848	1.163 (0.508–2.662)	0.721	0.743
BRCA1 rs1799966 × *BRCA2* rs15869	2.489 (1.035–5.983)	0.040 *	0.085
BRCA1 rs1799966 × *BRCA2* rs1799943	0.327 (0.140–0.763)	0.009 *	0.050
BRCA1 rs799917 × *BRCA1* rs8176318	10.675 (1.069–106.593)	0.015 *	0.050
BRCA1 rs799917 × *BRCA2* rs144848	0.710 (0.309–1.630)	0.419	0.628
BRCA1 rs799917 × *BRCA2* rs15869	1.897 (0.791–4.553)	0.150	0.245
BRCA1 rs799917 × *BRCA2* rs1799943	0.528 (0.226–1.232)	0.139	0.245
BRCA1 rs8176318 × *BRCA2* rs144848	1.149 (0.501–2.631)	0.743	0.743
BRCA1 rs8176318 × *BRCA2* rs15869	2.461 (1.025–5.910)	0.042 *	0.085
BRCA1 rs8176318 × *BRCA2* rs1799943	0.358 (0.154–0.835)	0.017 *	0.050
BRCA2 rs144848 × *BRCA2* rs15869	0.838 (0.350–2.005)	0.692	0.743
BRCA2 rs144848 × *BRCA2* rs1799943	1.297 (0.553–3.041)	0.550	0.743
BRCA2 rs15869 × *BRCA2* rs1799943	1.243 (0.501–3.079)	0.640	0.743

Ratio of ORs, exponentiated coefficient of the multiplicative interaction term in an age-adjusted logistic regression model including both main effects; SNPs were coded log-additively (0, 1, or 2 variant alleles) or dominantly. *p* values were obtained by likelihood ratio tests (LRT, 1 degree of freedom); *q*, false discovery rate across estimable tests within each coding. ‡ NE, not estimable because of quasi-complete separation arising from sparse discordant genotype categories between SNPs in strong linkage disequilibrium. Interaction terms for *BRCA1* SNP pairs within the same linkage disequilibrium block mainly reflect haplotype structure and should be interpreted with the haplotype analysis (Table S6). A statistical interaction on the multiplicative scale does not by itself indicate biological interaction.

Table S5. Association between *BRCA2* rs1799943 (GA + AA vs. GG) and RIF stratified by *BRCA1* genotype.

BRCA1 genotype stratum	Controls (GG/GA + AA)	RIF (GG/GA + AA)	AOR (95 % CI)	p
***BRCA1* rs12516 G>A**				
GG	70/120	27/34	0.756 (0.420–1.362)	0.352
GA + AA	32/108	38/29	0.227 (0.121–0.425)	< 0.001 *
***BRCA1* rs1799966 T>C**				
TT	64/112	28/36	0.758 (0.422–1.361)	0.354
TC + CC	38/116	37/27	0.241 (0.130–0.448)	< 0.001 *
***BRCA1* rs799917 G>A**				
GG	66/123	32/34	0.578 (0.327–1.023)	0.060
GA + AA	36/105	33/29	0.309 (0.165–0.581)	< 0.001 *
***BRCA1* rs8176318 C>A**				
CC	61/110	27/35	0.735 (0.406–1.331)	0.309
CA + AA	41/118	38/28	0.261 (0.142–0.478)	< 0.001 *

Numbers of participants with the rs1799943 GG and GA + AA genotypes are shown for each stratum. AOR (adjusted for age) compares rs1799943 GA + AA with GG within each *BRCA1* genotype stratum. *p* values for the corresponding interaction terms are given in Table S4.

Table S6. *BRCA1* haplotype analysis (rs12516–rs1799966–rs799917–rs8176318).

Haplotype	Controls (2n = 660)	RIF (2n = 256)	AOR (95 % CI)	p	q
G-T-G-C	447 (67.7)	172 (67.2)	1.000 (reference)		
A-C-A-A	134 (20.3)	71 (27.7)	1.270 (0.913–1.767)	0.156	0.295
A-C-G-A	28 (4.2)	6 (2.4)	0.653 (0.295–1.448)	0.295	0.295
G-T-A-C	17 (2.6)	2 (0.8)	0.417 (0.106–1.648)	0.213	0.295
G-C-A-A	12 (1.8)	0 (0.0)	NE		

Data are presented as the estimated number of chromosomes (%), derived from expectation–maximization frequency estimates in each group; haplotypes with a frequency ≥ 1 % in either group are shown (combined frequency of rarer haplotypes: 3.4 % in controls and 2.0 % in RIF). AOR per copy (adjusted for age) from haplotype-based logistic regression (haplo.glm), relative to G-T-G-C, which carries the major allele at all loci. NE, not estimable because the haplotype was not observed in one group. Global score test *p* = 0.066 (permutation *p* = 0.042).

Table S7. *BRCA2* haplotype analysis (rs144848–rs15869–rs1799943).

Haplotype	Controls (2n = 660)	RIF (2n = 256)	AOR (95 % CI)	p	q
A-A-G	173 (26.2)	88 (34.3)	1.000 (reference)		
A-A-A	262 (39.6)	68 (26.6)	0.551 (0.376–0.807)	0.002 *	0.012 *
C-A-G	91 (13.8)	36 (14.2)	0.848 (0.517–1.391)	0.514	0.877
A-C-G	68 (10.2)	31 (12.1)	0.958 (0.555–1.652)	0.877	0.877
C-C-G	41 (6.2)	22 (8.6)	1.143 (0.611–2.141)	0.675	0.877
C-A-A	19 (2.8)	11 (4.2)	0.923 (0.421–2.025)	0.842	0.877

Haplotype	Controls (2n = 660)	RIF (2n = 256)	AOR (95 % CI)	*p*	*q*
A-C-A	7 (1.0)	0 (0.0)	NE		

Data are presented as the estimated number of chromosomes (%), derived from expectation–maximization frequency estimates in each group; all haplotypes with a frequency ≥ 1 % in either group are shown. AOR per copy (adjusted for age) from haplotype-based logistic regression (haplo.glm), relative to A-A-G, which carries the major allele at all loci. NE, not estimable because the haplotype was not observed in one group. Global score test $p = 0.024$ (permutation $p = 0.025$).

Table S8. Complete results of the seven-locus allele-combination analysis.

Allele combination	Controls (2n = 660)	RIF (2n = 256)	AOR (95 % CI)	*p*	*q*
G-T-G-C-A-A-G	134 (20.2)	58 (22.5)	1.000 (reference)		
G-T-G-C-A-A-A	157 (23.8)	53 (20.8)	0.891 (0.525–1.512)	0.669	0.761
A-C-A-A-A-A-A	79 (11.9)	11 (4.3)	0.387 (0.169–0.884)	0.025 *	0.091
G-T-G-C-C-A-G	62 (9.4)	23 (9.0)	1.013 (0.503–2.037)	0.972	0.972
G-T-G-C-A-C-G	52 (7.8)	13 (5.2)	0.675 (0.277–1.642)	0.386	0.708
G-T-G-C-C-C-G	25 (3.8)	16 (6.4)	1.773 (0.725–4.334)	0.210	0.577
A-C-A-A-A-A-G	19 (2.9)	27 (10.6)	3.289 (1.247–8.676)	0.017 *	0.091
A-C-A-A-C-A-G	15 (2.3)	9 (3.6)	1.609 (0.585–4.425)	0.357	0.708
G-T-G-C-C-A-A	13 (2.0)	8 (3.3)	1.274 (0.500–3.249)	0.612	0.761
A-C-G-A-A-A-A	13 (1.9)	0 (0.0)	NE		
A-C-A-A-A-C-G	10 (1.5)	16 (6.1)	3.792 (1.328–10.825)	0.013 *	0.091
A-C-G-A-A-A-G	8 (1.3)	2 (0.9)	1.615 (0.221–11.798)	0.637	0.761
A-C-A-A-C-C-G	7 (1.0)	6 (2.3)	1.510 (0.197–11.600)	0.692	0.761

Allele combinations are listed in the order *BRCA1* rs12516–rs1799966–rs799917–rs8176318/*BRCA2* rs144848–rs15869–rs1799943. Because *BRCA1* and *BRCA2* are located on different chromosomes, these are statistically inferred multilocus allele combinations rather than physical haplotypes. They were estimated by applying the expectation–maximization algorithm to the unphased seven-locus genotypes under the assumption of Hardy–Weinberg equilibrium; because the two genes segregate independently, the phase inferred between them has no physical meaning, and these exploratory results do not indicate a biological mechanism or a synergistic effect between *BRCA1* and *BRCA2*. Data are presented as the estimated number of chromosomes (%), derived from expectation–maximization frequency estimates in each group; combinations with a frequency ≥ 1 % in either group are shown (combined frequency of rarer combinations: 10.0 % in controls and 5.1 % in RIF). AOR per copy (adjusted for age) from haplotype-based logistic regression (haplo.glm), relative to G-T-G-C-A-A-G, which carries the major allele at all loci. NE, not estimable because the haplotype was not observed in one group. Global score test $p < 0.001$ (permutation $p = 0.002$).
